# Bridging GNSS Outages with IMU and Odometry: A Case Study for Agricultural Vehicles

**DOI:** 10.3390/s21134467

**Published:** 2021-06-29

**Authors:** Eva Reitbauer, Christoph Schmied

**Affiliations:** Institute of Geodesy, Graz University of Technology, 8010 Graz, Austria; schmied@tugraz.at

**Keywords:** multi-sensor fusion, autonomous agricultural vehicles, Kalman filtering, autonomous compost turner, GNSS interference mitigation

## Abstract

Nowadays, many precision farming applications rely on the use of GNSS-RTK. However, when it comes to autonomous agricultural vehicles, GNSS cannot be used as a stand-alone system for positioning. To ensure high availability and robustness of the positioning solution, GNSS-RTK must be fused with additional sensors. This paper presents a novel sensor fusion algorithm tailored to tracked agricultural vehicles. GNSS-RTK, an IMU and wheel speed sensors are fused in an error-state Kalman filter to estimate position and attitude of the vehicle. An odometry model for tracked vehicles is introduced which is used to propagate the filter state. By using both IMU and wheel speed sensors, specific motion characteristics of tracked vehicles such as slippage can be included in the dynamic model. The presented sensor fusion algorithm is tested at a composting site using a tracked compost turner. The sensor measurements are recorded using the Robot Operating System (ROS). To analyze the achievable accuracies for position and attitude of the vehicle, a precise reference trajectory is measured using two robotic total stations. The resulting trajectory of the error-state filter is then compared to the reference trajectory. To analyze how well the proposed error-state filter is suited to bridge GNSS outages, GNSS outages of 30 s are simulated in post-processing. During these outages, the vehicle’s state is propagated using the wheel speed sensors, IMU, and the dynamic model for tracked vehicles. The results show that after 30 s of GNSS outage, the estimated horizontal position of the vehicle still has a sub-decimetre accuracy.

## 1. Introduction

In the last decade, Global Navigation Satellite Systems (GNSS) have played an important role in precision agriculture. GNSS positions are used to georeference data collected on fields. This includes soil samples, information on how much fertilizer is applied, or crop scouting, irrigation, and harvesting [1].

One of the most important GNSS applications in the agricultural sector is automatic steering. According to [2], automatic steering belongs to the main revenue-generating applications in the agricultural sector of the global GNSS downstream market. In [3] it is stated that automatic steering belongs to the fastest-growing segments of GNSS applications in the agricultural sector.

Automatic steering systems have already been developed for tractors, self-propelled sprayers, or harvesters. They offer many benefits, one of them being the possibility to continuously operate during time-critical farming operations such as harvesting. An overview of studies comparing the effects of automatic steering with manually-guided farming operations is given in [4].

When large agricultural machines are steered automatically, it is crucial to determine their position and attitude with high accuracy and reliability. Pini et al. [5] state that if the machine has poor positioning capabilities, it is unable to perform path-following. Typically, sub-decimetre accuracy is needed to steer agricultural machines [1], depending on the exact application. This accuracy requirement can be fulfilled by using GNSS-RTK.

However, GNSS outages are always possible, be it through intentional or unintentional interference. To overcome periods of GNSS outages, GNSS-RTK must be fused with other sensors. Most commonly, GNSS is fused with Inertial Navigation Systems (INS), see e.g., in [6,7]. To overcome longer periods of GNSS outages, high performance inertial sensors are needed. Zhang et al. [8] state that automotive-grade INS show a position drift of approximately one metre after a minute of GNSS outage, whereas low-cost MEMS Inertial Measurement Units (IMU) cause position errors of more than tens of metres per minute.

Han et al. [9] summarize recent developments for automatic steering systems in the agricultural sector. It analyzes that in addition to fusing GNSS with INS, vision-based sensors, tactile sensors, or scanners are often used as a backup or alternative to GNSS-based guidance. However, these systems also have drawbacks. When it comes to vision-based systems, illumination variation or cluttered backgrounds in open fields can lead to problems. Tactile sensors or scanners can only be used when crop rows are clearly defined.

While automatic steering systems are already used in tractors and harvesters, to the authors’ knowledge, no such system has yet been developed for compost turners. To understand why such a system would offer tremendous benefits for the composting sector, a brief overview of composting is given in the following.

Composting is a form of organic waste treatment where the waste is turned into carbon dioxide, ammonia, water, and humic substances [10]. Compost, the final product of this process, is biochemically stable and contains microorganisms. When added to agricultural soils, the organic matter content and fertility of the soil are increased.

In windrow composting, the most common method of commercial composting [11], the organic waste to be composted is stacked into long, triangle-shaped heaps called windrows. These windrows need to be turned regularly by compost turners to ensure aeration. The turning frequency may range from daily to weekly [12] and depends on the environmental conditions and age of the windrows. A typical turning schedule can consist of three turnings per week in the first two weeks for composting, two turnings in the third week, and one turning each in the fourth and fifth week [11]. Since operating compost turners is a monotonous and tedious task that has to be repeated on a regular basis, it would be a great benefit if compost turners could be steered automatically.

When comparing compost turners to tractors or harvesters, several differences can be noted. First, the driving speed of compost turners is much lower than those of tractors or harvesters. While the AmericanSocietyofAgriculturalandBiologicalEngineers [13] suggests a combine field speed between 3.0 and 6.5 km/h for harvesters, and Grisso et al. [14] state that the optimal field travel speed is between 6.4 and 9.7 km/h for tractors, compost turners operate at a much lower speed of less than 1 km/h. Moreover, compost turners vibrate heavily as they turn the windrows with their spiked drums. The low operating speed and strong vibrations make it difficult to propagate the vehicle’s state using IMUs. Additionally, most compost turners are tracked vehicles and are therefore steered differently than tractors or harvesters. Due to these reasons, an automatic steering system developed for compost turners differs from conventional automatic steering systems for tractors or harvesters.

In [15], we first proposed a concept for an automatic steering module for compost turners. In [16] we selected a set of navigation sensors for the positioning of compost turners and presented a concept for sensor fusion. Until now, no results of the field tests of the developed navigation module have been published. The present article intends to fill this gap.

This paper presents a positioning algorithm for tracked compost turners that fuses observations of a dual-antenna GNSS receiver, a mid-range MEMS IMU, and rotary encoders to estimate the position and attitude of the tracked vehicle. The developed extended Kalman filter is tested at a real composting site in Austria. During the field tests, all sensor measurements are recorded using the Robot Operating System (ROS) so that they can later be replayed for real-time simulations. To evaluate the achievable accuracies, a highly precise reference trajectory is measured. Two total stations are used to track two 360°-prisms that are mounted on the compost turner. The estimated position of the developed positioning algorithm is then compared to the reference trajectory.

To analyze how well the proposed filtering architecture is suited to overcome GNSS outages, GNSS outages of 30 s are simulated in post-processing. During these outages, the vehicle’s state is propagated using the measurements of the wheel speed sensors, a dynamic model for tracked vehicles, and the IMU. The main contributions of this paper are:

1. A novel approach for filtering GNSS, IMU, and wheel speed sensors for tracked vehicles. An error state Kalman filter is used to fuse the measurements. In contrast to conventional systems where the IMU is used as a reference sensor (see [17,18,19,20,21]), here the wheel speed sensors, a yaw rate, and a dynamic model for tracked vehicles are used to propagate the filter state. This allows to propagate the vehicle’s state with higher accuracy at low dynamic applications.
2. For the first time, a positioning module tailored to compost turners is tested at a real composting site. By comparing the estimated positions to a highly precise reference trajectory, the achievable accuracy of the positioning module can be analyzed.
3. GNSS outages are simulated in post-processing and bridged by the dynamic model using wheel speed sensors. The performance of the filter during GNSS outages is analyzed by comparing the state estimates to the reference trajectory.

The paper is organized as follows: Section 2 presents the materials an methods, where Section 2.1 gives a brief overview of the used sensors, Section 2.2 presents the filtering architecture, Section 2.3 describes the software development, and Section 2.4 presents the test setup. In Section 3, the achievable accuracies for the position and heading with and without GNSS outages are presented. Section 4 discusses the results and concludes the paper.

## 2. Materials and Methods

### 2.1. Selected Navigation Sensors

The assortment of sensors used in this study consists of a geodetic-grade dual-antenna GNSS receiver, a mid-range MEMS IMU, and two rotary encoders for odometry information. The GNSS receiver is an Alberding A12-RTK which internally consists of a Trimble BD992 dual-antenna module providing precise position and heading. Additionally, the receiver contains an LTE modem which makes it capable to process network RTK corrections in real-time. The network RTK provider used in this study is EPOSA, an Austrian company providing a nationwide network of GNSS reference stations. The antennas used for the GNSS receiver are two Trimble GA810, offering quality signal tracking and high multipath rejection and supporting GALILEO, GPS, and GLONASS. The IMU is a mid-range MEMS sensor by the company XSens. The tracks of the vehicle used in this study can be steered independently and are controlled by electric motors in combination with inverters acting as the control modules. These modules are equipped with rotary encoders providing feedback for the motor control. Information about the wheel speed is gained by reading encoder data available on the CAN bus of the vehicle. A summary of the selected sensors is stated in Table 1.

To get from rotations per second of the rotary encoder to the speed of a track, the following considerations have to be taken into account:

- The diameter of the track wheel plus the thickness of the track have to be known precisely;
- The motor drives the track wheel via a gear ratio of 79.5:1.

Taking these considerations into account, the speed of a track $v_{track}$ in [m/s] can be calculated by:

(1) $$v_{track}=\frac{v_{r}}{79.5}\cdot d\pi ,$$

where $v_{r}$ is the radial velocity of the track wheel in Hz and *d* is the overall diameter of the track segment.

### 2.2. Extended Kalman Filtering

To estimate position and attitude of the vehicle, we use a Kalman filter. When the functional relationship of the dynamic model and the observation model are nonlinear, they need to be linearized and we speak of extended Kalman filtering [22]. In the following, we explain the steps of the Kalman filter, where we use a hat (^) to indicate an estimated variable and a tilde (~) for predicted variables.

In the time update or prediction step of the filter, the estimated error state vector $\delta {\hat{x}}_{k-1}$ of the previous epoch k−1 and its covariance matrix $P_{k-1}$ are predicted to the current epoch *k* as follows:

(2) $$\delta {\tilde{x}}_{k}=\Phi_{k-1}\delta {\hat{x}}_{k-1},$$

(3) $${\tilde{P}}_{k}=\Phi_{k-1}P_{k-1}\Phi_{k-1}^{T}+Q_{k-1},$$

where $\Phi_{k-1}$ it the linearized transition matrix evaluated at Taylor point ${\hat{x}}_{k-1}$, and $Q_{k-1}$ is the system noise matrix.

In the next step, the so-called gain computation, the Kalman weight matrix $K_{k}$ is calculated using the linearized design matrix $H_{k}$, the covariance matrix of the observations $R_{k}$, as well as the predicted covariance matrix ${\tilde{P}}_{k}$ of the state vector in the following form:

(4) $$K_{k}={\tilde{P}}_{k}H_{k}^{T}{\left(H_{k}{\tilde{P}}_{k}H_{k}^{T}+R_{k}\right)}^{-1}.$$

The Kalman weight matrix is then used in the measurement update. The matrix expresses how much weight is given to the new observations to update the state in comparison to the prediction.

For the measurement update, the reduced observations $\delta z_{k}$ are computed from:

(5) $$\delta z_{k}=H_{k}\delta {\tilde{x}}_{k}-z_{k},$$

where the design matrix $H_{k}$ is evaluated at the Taylor point ${\tilde{x}}_{k}$ and $z_{k}$ are the observations.

We can now compute the measurement update from:

(6) $$\delta {\hat{x}}_{k}=K_{k}\delta z_{k},$$

(7) $$P_{k}=\left(I-K_{k}H_{k}\right){\tilde{P}}_{k}.$$

#### Fusing GNSS, IMU, and Odometry

To fuse observations of GNSS, the IMU, and wheel speed sensors, we use an error state Kalman filter. We define the state vector x as:

(8) $$x=\left(\begin{array}{c} \varphi \\ \lambda \\ h\\ \theta \\ \phi \\ \psi \end{array}\right),$$

where φ,λ, and *h* are the ellipsoidal coordinates of the vehicle, and θ,ϕ,ψ are the attitude parameters roll,pitch, and yaw. The error state vector δx is defined in a local level frame (North-East-Down, NED):

(9) $$\delta x=\left(\begin{array}{c} \delta N\\ \delta E\\ \delta D\\ \delta \theta \\ \delta \phi \\ \delta \psi \end{array}\right).$$

This implies that when the estimated error state vector is used to update the state, the position corrections in the NED-frame must be converted to an ellipsoidal frame. The state vector is therefore updated as follows:

(10) $$x_{new}={\left(\begin{array}{c} \varphi \\ \lambda \\ h\\ \theta \\ \phi \\ \psi \end{array}\right)}_{old}+\left(\begin{array}{c} \delta N/(M_{ell}+h)\\ \delta E/[\left(N_{ell}+h\right)\cdot \cos\varphi ]\\ -\delta D\\ \delta \theta \\ \delta \phi \\ \delta \psi \end{array}\right),$$

with $N_{ell}$ being the radius of curvature of the ellipsoid in the prime vertical plane and $M_{ell}$ being the radius of curvature in the meridian [19].

**Figure 1:**
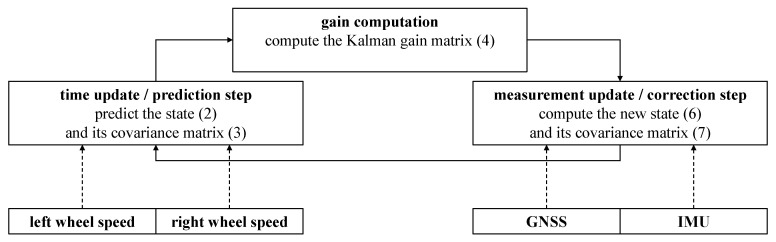
Schematic overview of the Kalman filter that fuses GNSS, IMU, and wheel speed sensors. The wheel speeds are used in the prediction step of the filter, GNSS, and IMU observations are used in the correction step.

Figure 1 gives an overview of how the sensor measurements are introduced to the Kalman filter. The left and right wheel speeds are used in the time update, where the state and its covariance matrix are predicted to the next epoch (see Formulas (2) and (3)). The preprocessed GNSS and IMU observations are used in the measurement update to correct the state and its covariance matrix (see Formulas (6) and (7)).

In the following, we explain in detail how the updates are computed for the individual sensors.

**Time Update with Wheel Speed Sensors**

For error state Kalman filters, where the error in the state vector rather than the full state vector is estimated, we apply a linear system model [18] in the following form:

(11) $$\delta \dot{x}\left(t\right)=F\left(t\right)\delta x\left(t\right)+G\left(t\right)w_{s}\left(t\right).$$

Here, F is the system matrix, G is the system noise distribution matrix, and $w_{s}$ is the system noise vector. In the time update of the Kalman filter, we need to compute the transition matrix Φ. This is done by computing a power-series expansion of the system matrix F for the propagation interval τ:

(12) $$\Phi_{k-1}=I+F_{k-1}\tau +\frac{F^{2}}{2}\tau^{2}+\frac{F^{3}}{6}\tau^{3}.$$

The system matrix F:

(13) $$F_{k-1}={\left.\frac{\partial f(x)}{\partial x}\right|}_{x={\hat{x}}_{k-1}}$$

contains the derivatives of the system model describing the dynamics of the vehicle. The differential equations $\dot{x}=f\left(x\right)$ can be obtained by using odometry. However, when using odometry for tracked vehicles on loose slopes, we need to take slippage into account [23]. For the tracked agricultural vehicle, we only model longitudinal slippage and neglect lateral slippage, as when the vehicle’s velocity is low enough and the lateral friction force is high enough, lateral slippage is almost zero [24]. We therefore adapt the differential equations described in [25] to describe the changes in ellipsoidal position ($\dot{\varphi },\dot{\lambda }$) and heading $\dot{\psi }$:

(14) $$\dot{\varphi }=\frac{1}{M_{ell}+h}\cdot \frac{v_{r}\left(1-a_{r}\right)+v_{l}\left(1-a_{l}\right)}{2}\cos\left(\psi \right),$$

(15) $$\dot{\lambda }=\frac{1}{(N_{ell}+h)\cos\varphi }\cdot \frac{v_{r}\left(1-a_{r}\right)+v_{l}\left(1-a_{l}\right)}{2}\sin\left(\psi \right),$$

(16) $$\dot{\psi }=\frac{v_{r}\left(1-a_{r}\right)-v_{l}\left(1-a_{l}\right)}{2d}.$$

**Figure 2:**
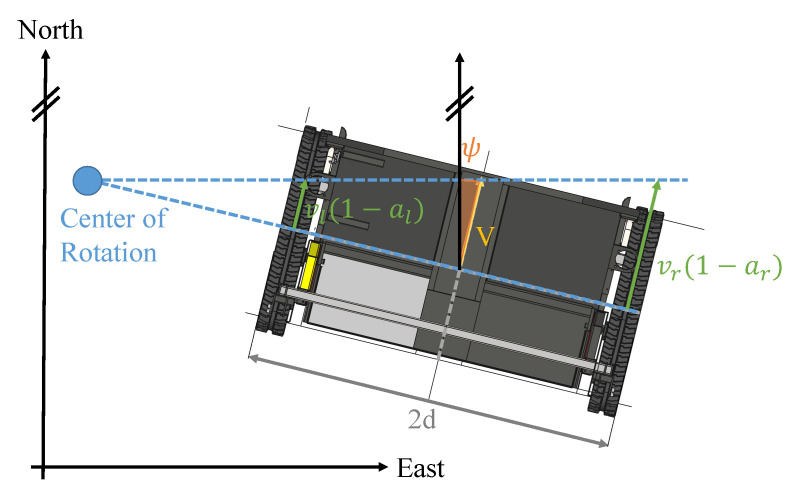
Dynamic model for tracked agricultural vehicles.

In Equations (14)–(16), $v_{r}$ and $v_{l}$ are the velocities measured by the wheel speed sensors, $a_{r}$ and $a_{l}$ are the slip ratios, and 2d is the track width of the vehicle. Again, $N_{ell}$ is the radius of curvature of the ellipsoid in the prime vertical plane and $M_{ell}$ is the radius of curvature in the meridian [19]. Note that the dynamic model only considers changes in horizontal position and heading, and neglects height changes and changes in roll and pitch. Figure 2 shows the dynamic model described in Equations (14)–(16).

The slip ratios are defined as:

(17) $$a_{r}=\frac{v_{r}-v_{r}^{\prime }}{v_{r}},$$

(18) $$a_{l}=\frac{v_{l}-v_{l}^{\prime }}{v_{l}},$$

where $v_{r}$ and $v_{l}$ are the measured speeds and $v_{r}^{\prime }$ and $v_{l}^{\prime }$ are the ground speeds. When no slippage occurs, the measured speeds by the wheel speed sensors are equal to the ground speeds and the slip ratios are zero.

When a yaw rate $\tilde{\dot{\psi }}$ is measured (e.g., by an IMU), Equation (16) can be used to compute the slip ratios, if an additional constraint is introduced. Therefore, we adopt a method proposed by [24] that introduces the following condition for the slip ratios:

(19) $$a_{r}=-\mathrm{sgn}\left(v_{r}\cdot v_{l}\right)a_{l},$$

where sgn is the signum function. When both tracks rotate in opposite direction, they generate a traction force and cause a positive slip ratio. When the tracks rotate in the same direction, the faster track generates a traction force and positive slip ratio, the slower track causes a breaking force and a negative slip ratio [24]. By inserting Equation (19) into (16) we can compute the slip ratios as:

(20) $$a_{r}=\frac{v_{r}-v_{l}-2d\tilde{\dot{\psi }}}{v_{r}+\mathrm{sgn}\left(v_{r}\cdot v_{l}\right)v_{l}},$$

(21) $$a_{l}=\frac{v_{l}-v_{r}+2d\tilde{\dot{\psi }}}{v_{r}+\mathrm{sgn}\left(v_{r}\cdot v_{l}\right)v_{l}}.$$

The system matrix F (Formula (13)) for our state vector and dynamic model has the following form:

(22) $$F=\left(\begin{array}{cccccc} 0 & 0 & 0 & 0 & 0 & \frac{\partial \dot{\varphi }}{\partial \psi }\\ 0 & 0 & 0 & 0 & 0 & \frac{\partial \dot{\lambda }}{\partial \psi }\\ 0 & 0 & 0 & 0 & 0 & 0\\ 0 & 0 & 0 & 0 & 0 & 0\\ 0 & 0 & 0 & 0 & 0 & 0\\ 0 & 0 & 0 & 0 & 0 & 0 \end{array}\right),$$

where all derivatives except for $\frac{\partial \dot{\varphi }}{\partial \psi }$ and $\frac{\partial \dot{\lambda }}{\partial \psi }$ are zero. By inserting:

(23) $$\frac{\partial \dot{\varphi }}{\partial \psi }=\frac{1}{M_{ell}+h}\cdot \frac{v_{r}\left(1-a_{r}\right)+v_{l}\left(1-a_{l}\right)}{2}\cdot -\sin\left(\psi \right)$$

and

(24) $$\frac{\partial \dot{\lambda }}{\partial \psi }=\frac{1}{(N_{ell}+h)\cdot \cos\varphi }\cdot \frac{v_{r}\left(1-a_{r}\right)+v_{l}\left(1-a_{l}\right)}{2}\cdot \cos\left(\psi \right)$$

into Formula (22), we can calculate the transition matrix Φ using Formula (12). In the next step, the system noise matrix Q is computed through a variance propagation:

(25) $$Q=J\cdot \left(\begin{array}{cc} \sigma_{v_{r}}^{2} & 0\\ 0 & \sigma_{v_{l}}^{2} \end{array}\right)\cdot J^{T},$$

where $\sigma_{v_{r}}^{2}$ and $\sigma_{v_{l}}^{2}$ are the variances of the wheel speed sensors (assumed to be uncorrelated) and J is defined as:

(26) $$J=\left(\begin{array}{cc} \frac{\partial \varphi }{\partial v_{r}} & \frac{\partial \varphi }{\partial v_{l}}\\ \frac{\partial \lambda }{\partial v_{r}} & \frac{\partial \lambda }{\partial v_{l}}\\ 0 & 0\\ 0 & 0\\ 0 & 0\\ \frac{\partial \psi }{\partial v_{r}} & \frac{\partial \psi }{\partial v_{l}} \end{array}\right).$$

As mentioned earlier, the differential equations of the dynamic model (Formulas (14)–(16)) only considers horizontal position changes and heading changes and neglects changes in height, roll, and pitch. To model these uncertainties, we introduce three additional variances $\sigma_{h}^{2}$, $\sigma_{\phi }^{2}$, $\sigma_{\theta }^{2}$ for height, roll, and pitch, and add them to the system noise matrix:

(27) $$\bar{\Phi }=\Phi +\left(\begin{array}{cccccc} 0 & 0 & 0 & 0 & 0 & 0\\ 0 & 0 & 0 & 0 & 0 & 0\\ 0 & 0 & \sigma_{h}^{2} & 0 & 0 & 0\\ 0 & 0 & 0 & \sigma_{\phi }^{2} & 0 & 0\\ 0 & 0 & 0 & 0 & \sigma_{\theta }^{2} & 0\\ 0 & 0 & 0 & 0 & 0 & 0 \end{array}\right).$$

**Measurement Update with GNSS Dual-Antenna Array**

With the GNSS dual-antenna array, ellipsoidal coordinates φ, λ, and *h*, as well as the heading angle ψ can be observed and introduced as observations into the Kalman filter. When the GNSS position antenna is not located at the centre of the vehicle, a lever arm correction must be applied. The known lever arm in the body frame of the vehicle $l_{b}$ can be rotated to the local level frame (NED-frame):

(28) $$l_{l}=\left(\begin{array}{c} l_{N}\\ l_{E}\\ l_{D} \end{array}\right)=R_{b}^{l}\cdot l_{b}$$

with the rotation matrix:

(29) $$R_{b}^{l}=\left(\begin{array}{ccc} \cos\theta \cos\psi & \sin\phi \sin\theta \cos\psi -\cos\phi \sin\psi & \cos\phi \sin\theta \cos\psi +\sin\phi \sin\psi \\ \cos\theta \sin\psi & \sin\phi \sin\theta \sin\psi +\cos\phi \cos\psi & \cos\phi \sin\theta \sin\psi -\sin\phi \cos\psi \\ -\sin\theta & \sin\phi \cos\theta & \cos\phi \cos\theta \end{array}\right).$$

We can compute the reduced observations $\delta z_{GNSS}$, which are needed to estimate the error state vector, in the NED-frame:

(30) $$\delta z_{GNSS}=\left(\begin{array}{c} \left(M_{ell}+\tilde{h}\right)\tilde{\varphi }-\left(\left(M_{ell}+\tilde{h}\right)\varphi_{GNSS}-l_{N}\right)\\ \left(N_{ell}+\tilde{h}\right)\cdot \cos\tilde{\varphi }\cdot \tilde{\lambda }-\left(\left(N_{ell}+\tilde{h}\right)\cdot \cos\tilde{\varphi }\cdot \lambda_{GNSS}-l_{E}\right)\\ \tilde{h}-h_{GNSS}+l_{D}\\ \tilde{\psi }-\psi_{GNSS} \end{array}\right).$$

The design matrix H has the following form:

(31) $$H=\left(\begin{array}{cc} I_{3x3} & A_{3x3}\\ 0_{1x3} & \left(\begin{array}{ccc} 0 & 0 & 1 \end{array}\right) \end{array}\right),$$

where A is the axiator matrix with respect to the lever arm in the NED-frame. With the design matrix in Formula (31) we can now compute the Kalman weight (Formula (4)). Then, the Kalman weight and the reduced observations (Formula (30)) are used to estimate the error state vector (Formula (6)). The error state vector is then used to update the state with Formula (10).

**Measurement Update with MEMS IMU**

In the measurement update with the IMU, we use the measured accelerations to compute roll and pitch. In a first step, the accelerations f must be brought from the sensor frame *s* to the body frame of the vehicle *b* through:

(32) $$f^{b}=\left(\begin{array}{c} acc_{x}\\ acc_{y}\\ acc_{z} \end{array}\right)=R_{s}^{b}\cdot f^{s},$$

where $R_{s}^{b}$ is the matrix describing the rotation from the sensor to the body frame. From the accelerations in the body frame we can now compute the attitude angles roll: (ϕ)

(33) $$\phi_{IMU}=\arctan\frac{-acc_{y}}{-acc_{z}},$$

and pitch (θ):

(34) $$\theta_{IMU}=\arctan\frac{acc_{x}}{\sqrt{acc_{y}^{2}+acc_{z}^{2}}}.$$

$\phi_{IMU}$ and $\theta_{IMU}$ are filtered with an Alpha-Beta-Filter before they are used to compute the reduced observations:

(35) $$\delta z_{IMU}=\left(\begin{array}{c} \tilde{\phi }-\phi_{IMU,filtered}\\ \tilde{\theta }-\theta_{IMU,filtered} \end{array}\right).$$

The design matrix H which is then used in the computation of the Kalman weight and to estimate the error state vector looks as follows:

(36) $$H=\left(\begin{array}{cccccc} 0 & 0 & 0 & 1 & 0 & 0\\ 0 & 0 & 0 & 0 & 1 & 0 \end{array}\right).$$

### 2.3. Software Development

**Figure 3:**
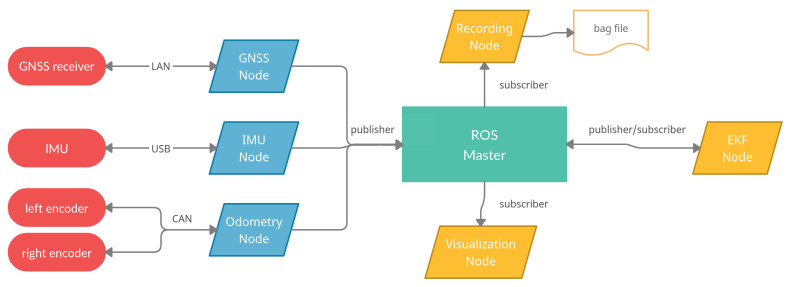
ROS program flow. Sensor hardware is depicted in red, sensor nodes in blue, subscriber nodes in yellow, and the ROS master in turquoise. The arrowheads indicate if the flow is one way or both ways.

All software development has been done in C++ using the ROS framework to be able to communicate with sensors and compute a solution in real-time. One of the advantages using ROS is that the sensor hardware can easily be decoupled from the overall program flow which is illustrated in Figure 3. Each sensor node (blue) acquires data by utilizing the sensor specific hardware interfaces (red). This data is afterwards packaged in a ROS message and transmitted to the ROS master using a publish-subscribe pattern. All subscribers in ROS (yellow) have callbacks for specific messages, for example a GNSS fix message. Therefore, the implementation of a data-driven Extended Kalman Filter (EKF) is easy to achieve because the update step in the filter is only executed when data is transmitted from a sensor node. Another advantage is that multiple nodes can subscribe to the same topic and process it at the same time. Thus it is possible to compute a solution, visualize current data, and record this data at the same time. Data recording is another advantage because by recording data into so-called bag files, it is possible to replay these files later and simulate a real-time environment. By utilizing callbacks for gaining sensor information, GNSS outages can easily be simulated by disabling the GNSS message publisher.

When multiple sensors are used, time synchronization is critical. In low dynamic applications with update rates of up to 100 Hz, the timestamp provided by the ROS master node is sufficient to synchronize all other nodes. If the application is highly dynamic, hardware timestamping has to be considered. In our study the compost turners dynamics are low enough that we use the ROS timestamp for synchronization purposes.

### 2.4. Field Tests

**Figure 4:**
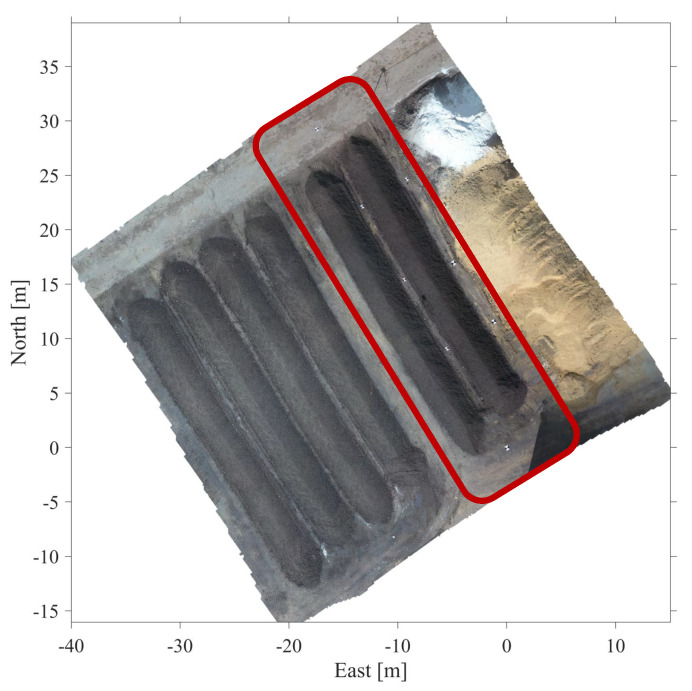
Orthophoto of region where the field tests were conducted. The windrows of the test area are circled in red. The orthophoto was generated from UAV photogrammetry by Viktor Kaufmann, Institute of Geodesy, Graz University of Technology. UAV flight by Gernot Seier, Department of Geography and Regional Science, University of Graz.

In March 2021, the developed positioning algorithm was tested at the composting site of Sonnenerde GmbH in Riedlingsdorf, Austria. Figure 4 shows an orthophoto of the test area. During the tests, a tracked compost turner drove through two windrows, which are shown in red in the photo. Both windrows have a length of approximately 30 m.

**Figure 5:**
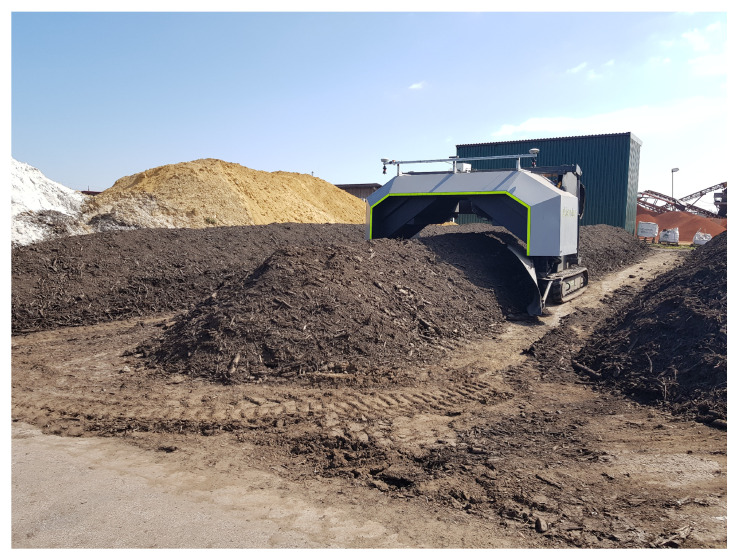
The tracked compost turner eWender E35eco during the field tests.

The tracked compost turner used for the field tests was the eWender E35eco by Pusch & Schinnerl GmbH. In contrast to conventional compost turners, which are usually diesel-driven, the eWender is electrically driven [26] and can be steered remotely. Figure 5 shows a picture of the eWender driving through the windrows during the field tests.

**Figure 6:**
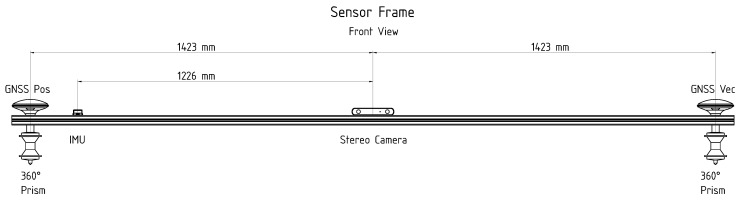
Mounting of the navigation sensors on the aluminum profile. The lever arms to the centre are shown in millimetres.

To collect data with navigation sensors, an aluminum profile was mounted on the tracked compost turner. In Figure 5 it can be seen that the aluminum profile was mounted on the across-axis of the machine. Figure 6 shows the front view of the aluminum profile. The two GNSS antennas (position and vector antenna) were mounted on the outer left and right side of the profile, to generate a baseline of 2.846 m. The IMU was placed next to the GNSS position antenna and a stereo camera (which was not used in the positioning algorithm of this study) was mounted at the centre. The wheel speed sensors are not shown as they are located inside the vehicle.

To generate a reference trajectory, two Leica 360°-prisms were also mounted on the profile, below the GNSS antennas. Two robotic total stations were used to track the prisms. The manufacturer’s specifications ([27,28]) for the achievable accuracies of angle and distance measurements are stated in Table 2.

The mounting of the 360°-prisms along the across-axis of the compost turner allows to generate a reference trajectory for position and heading. For a precise reference trajectory, the timestamps of the total station must be synchronized with the timestamps of the navigation sensors. To synchronize the two total stations with each other, the same 360°-prism was tracked while being lifted and set to the ground again. In post-processing, the resulting height time series of the tracking of the same prism were correlated to determine the offset of the total stations’ system times. To determine the offset between the total station system time and the system time of the navigation sensors, the coordinate time series of the GNSS position antenna were correlated with the coordinate time series of the tracked prism located below the GNSS position antenna.

## 3. Results

This section presents the results of the field tests conducted in March 2021. In Section 3.1, the computed roll and pitch angles from the accelerations measured by the IMU, as well as the track speeds computed from the rotary encoder readings, are analyzed. Section 3.2 presents the achievable accuracies of the proposed filter for horizontal position and heading in a scenario where all sensor data are available. Section 3.3 analyzes how the filter performs during simulated GNSS outages of 30 s.

### 3.1. Analysis of Filter Observations

**Figure 7:**
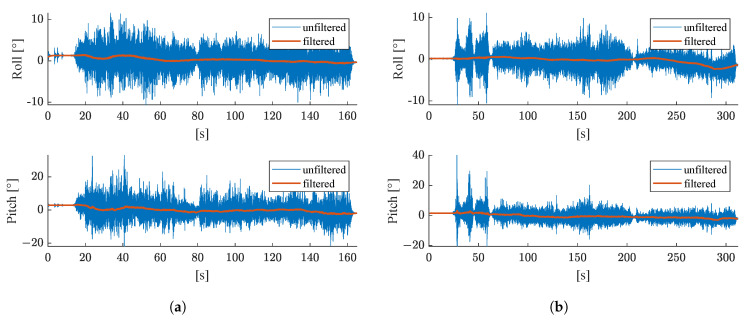
Unfiltered (blue) and filtered (orange) attitude angles roll and pitch computed from accelerations measured with the XSens MTi-G-710. For the filtered results, an Alpha-Beta-Filter with $\alpha =10^{-2}$ and $\beta =10^{-8}$ was used. Results for (**a**) the first test run, (**b**) the second test run.

In the proposed filtering algorithm, the accelerations measured by the MEMS IMU are used to compute the angles roll ϕ (see Formula (33)) and pitch θ (see Formula (34)). As the measurements are strongly affected by noise due to vibrations of the machine, the computed values for roll and pitch are filtered with an Alpha-Beta-Filter before they are fed into the filter. Figure 7 shows both the filtered and unfiltered results for the angles roll and pitch for both test runs. Without filtering, the noise for the roll angle is in the range of $\pm 10^{\circ }$, for the pitch angle it even ranges to $\pm 40^{\circ }$ in the second test run.

**Figure 8:**
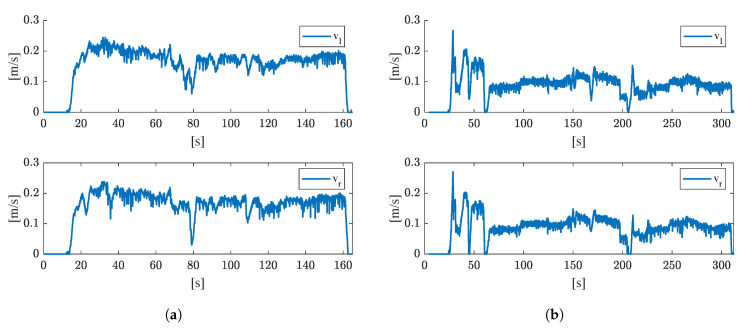
Left ($v_{l}$) and right ($v_{r}$) track speeds for (**a**) the first test run, and (**b**) the second test run. The track speeds are calculated according to Formula (1), where a diameter *d* = 38.5 [cm] is used.

To obtain the left and right track speeds which are used to propagate the vehicles’ state, the radial velocities measured by the rotary encoders are converted to track speeds with Formula (1). Figure 8 shows the left ($v_{l}$) and right ($v_{r}$) track speeds for both test runs. The left and right track speeds within the same test run hardly differ from each other, as the vehicle drove along a straight path in both test runs. When the speeds of the first (**a**) and second (**b**) test run are compared, it is noticeable that the vehicle drove faster during the first test run (approximately 0.2 [m/s]) than in the second (approximately 0.1 [m/s]).

### 3.2. Achievable Accuracies for Position and Heading without GNSS Outages

To analyze how accurately the position of the compost turner can be determined, the positions obtained from the Kalman filter are compared to the reference trajectory. The reference trajectory was generated through two 360°-prisms that were mounted on the compost turner. The prisms were tracked by two robotic total stations (see Section 2.4). Through a coordinate transformation, the positions estimated by the Kalman filter were brought to the same local level frame as the reference trajectory. The deviations from the reference trajectory were computed as follows:

(37) $$\Delta N\left(t\right)=N_{EKF}\left(t\right)-N_{ref}\left(t\right),$$

(38) $$\Delta E\left(t\right)=E_{EKF}\left(t\right)-E_{ref}\left(t\right),$$

(39) $$\Delta Hor\left(t\right)=\sqrt{\Delta N^{2}\left(t\right)+\Delta E^{2}\left(t\right)},$$

where $N_{EKF}\left(t\right)$ and $E_{EKF}\left(t\right)$ are the North and East coordinates estimated by the Kalman filter at epoch *t*, and $N_{ref}\left(t\right)$ and $E_{ref}\left(t\right)$ are the North and East coordinates of the reference trajectory at the same epoch *t*. ΔHor(t) is the horizontal positioning accuracy at epoch *t*.

**Figure 9:**
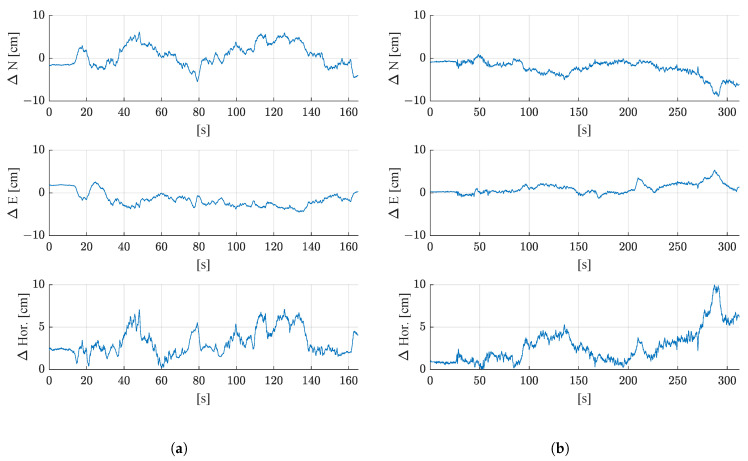
Deviation from the reference trajectory in north (ΔN), east (ΔE), and 2D horizontal deviation (ΔHor) for (**a**) the first test run, and (**b**) the second test run.

Figure 9 shows the achieved accuracies in the north, east, and horizontal positioning accuracy of the proposed filter for both test runs. The deviations of the estimated filter position from the reference trajectory as time series for the north, east, and horizontal position. In both test runs, the maximum deviation from the reference trajectory is below 10 cm.

The achievable accuracy for the heading is analyzed by computing the deviation of the estimated heading by the filter ($\psi_{EKF}$) from the reference heading ($\psi_{ref}$) as:

(40) $$\Delta \psi \left(t\right)=\psi_{EKF}\left(t\right)-\psi_{ref}\left(t\right).$$

**Figure 10:**
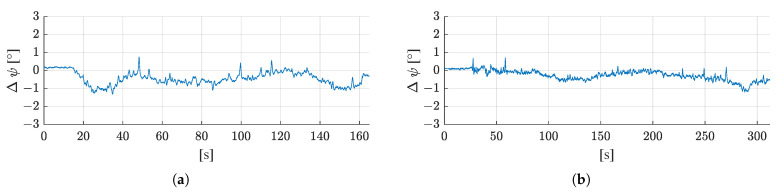
Deviation of the estimated heading (ψ) of the filter from the reference heading for (**a**) the first test run, and (**b**) the second test run.

Figure 10 shows how the estimated heading deviates from the heading of the reference trajectory in both test runs. The maximum deviation from the reference is 1.3° in the first test run and 1.2° in the second run.

The mean deviations from the reference trajectory, including the standard deviations of the difference time series, are shown in Table 3. In both test runs, a similar accuracy was achieved for the horizontal position estimate (3.2 cm in the first and 2.7 cm in the second test run) and heading (−0.4° in the first and −0.3° in the second test run).

### 3.3. Achievable Accuarcies for Position and Heading with GNSS Outages

**Figure 11:**
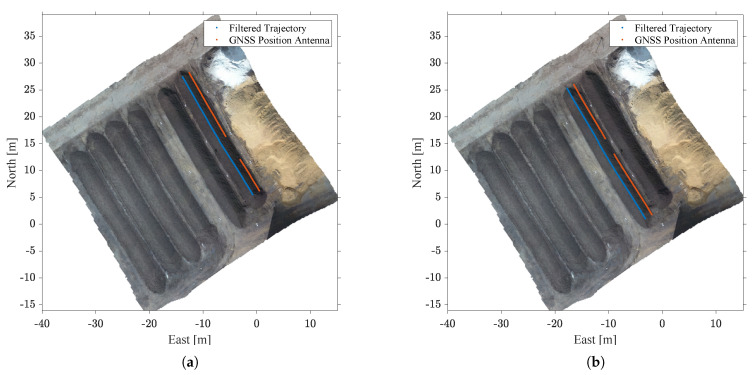
Simulated GNSS outages for (**a**) the first test run, and (**b**) the second test run. The available GNSS positions are shown in orange, the trajectory resulting from the Kalman filter is shown in blue.

To analyze how well the proposed filter is suited to bridge GNSS outages, random GNSS outages with a duration of 30 s were simulated in post-processing. Note that outage durations of 30 s were chosen since composting sites are often located next to motorways. In [29] the impact of GNSS jamming events on a receiver located close to a German motorway is analyzed. The study shows that the drop in the carrier-to-noise ratio of the receiver can last up to 30 s. Figure 11 shows how the GNSS outages were simulated for both test runs. The GNSS positions that were used as filter observations are shown in orange, whereas the filter result is shown in blue. Note that the GNSS positions have an offset of 1.423 m with respect to the filter result, as the GNSS position antenna was mounted on the left side of the vehicle (see Figure 6), and the filter estimates the position at the vehicles’ centre.

In Figure 11 it can be seen that the outage in the first test run affects a larger part of the trajectory than the outage in the second test run, which is to be expected as the driving speed was higher in the first test run (see Figure 8).

**Figure 12:**
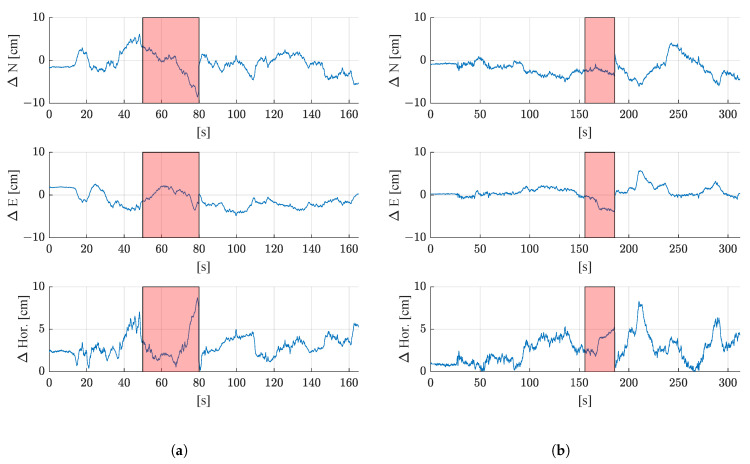
Deviation from the reference trajectory with simulated GNSS outages of 30 s in north (ΔN), east (ΔE), and 2D horizontal deviation (ΔHor) for (**a**) the first test run, and (**b**) the second test run. The epochs where no GNSS was available are shown in red.

Figure 12 shows the deviations of the estimated filter position from the reference trajectory as time series for the north, east, and horizontal positioning error. The periods where GNSS outages were simulated are shown in red. Similar to the results without GNSS outages (see Figure 9), the maximum deviation from the reference trajectory stays below 10 cm for both test runs. The maximum 2D deviation from the reference is 8.7 cm for the first test run and 8.3 cm for the second test run. When investigating the periods where GNSS outages were simulated more closely, it can be seen that the 2D positioning error grows with the duration of the outages. This is to be expected, since when only dead reckoning is used to propagate the state vector (as it is the case during GNSS outages), errors accumulate with time. The error grows faster for the first test run, which might be due to the fact that the average driving speed was higher. It might also be caused by a small turn of the vehicle, as shortly before second 80 of the first test run, the speeds of the right and left tracks differ (see Figure 8). However, this needs to be investigated more closely in further studies where the vehicle also follows curved paths.

**Figure 13:**
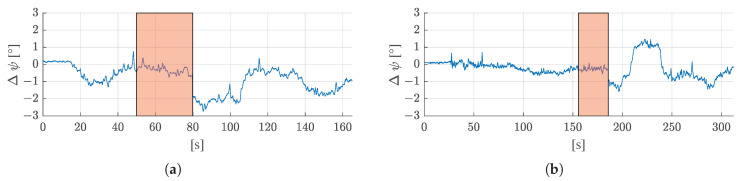
Deviation of the estimated heading (ψ) of the filter from the reference heading for (**a**) the first test run, and (**b**) the second test run. The red areas indicate the periods where GNSS outings were simulated.

Figure 13 shows the deviation of the estimated heading from the reference heading for both test runs with a simulated GNSS outage of 30 s each. Again, the red areas show where the GNSS outages were simulated. It can be seen that during the outages, the estimated heading hardly deviates from the reference heading. During the GNSS outages, the heading changes are only computed from Formula (16), where the left and right track speeds measured by the rotary encoders, as well as the yaw rate measured by the IMU (which is used to estimate the slip ratios) are used as observations. After the outages, the estimated heading is updated with the next available GNSS heading.

Table 4 shows how the filtered solutions with simulated 30-s GNSS outages deviate from the reference trajectory. The mean deviation as well as the standard deviations of the difference time series for north, east, horizontal position, and heading are shown. When comparing these values to the results without simulated GNSS outages (Table 3), it can be seen that the mean position errors are in a similar range. The mean horizontal error of the first round is 3.0 cm with a GNSS outage and without a GNSS outage it is 3.2 cm. The mean horizontal error of the second round is 2.6 cm with GNSS outage, without simulated outage it is 2.7 cm. The heading error is lower when no GNSS outages occur (−0.4° with a standard deviation of 0.4° for the first test run, and −0.3 cm° with a standard deviation of 0.3° for the second test run) than when 30-s GNSS outages are simulated (mean error of 0.8° with a standard deviation of 0.7° for the first round, mean error of −0.2° with a standard deviation of 0.6° for the second round).

## 4. Discussion

This paper investigated how GNSS outages can be bridged with IMU and odometry for tracked agricultural vehicles. Most automatic steering systems in the agricultural sector heavily rely on GNSS. When GNSS outages occur, short outages are bridged with automotive-grade INS. With low-cost MEMS IMUs, the position starts to drift rapidly when GNSS is unavailable.

In this study, we proposed a novel approach to filter observations of a dual-antenna GNSS receiver, a MEMS IMU, and rotary encoders to estimate position and attitude of a tracked compost turner using an error state Kalman filter. Contrary to conventional error state filters, which use an IMU to propagate the state, we presented a dynamic model for tracked vehicles that accounts for longitudinal slip and uses track speeds measured by rotary encoders and a yaw rate measured by an IMU to propagate the vehicle’s state.

The proposed filter to the estimate position and attitude of the tracked compost turner was tested at a composting site in Riedlingsdorf, Austria. An electrically-driven, tracked compost turner was equipped with two GNSS antennas, a GNSS receiver, and a MEMS IMU. Data measured by the rotary encoders of the vehicle were accessed via a CAN interface. Additionally, two 360°-prisms were mounted on the vehicle and tracked by two robotic total stations to generate a reference trajectory. The estimated horizontal position and heading were later compared to the reference trajectory to analyze which accuracy can be achieved with the proposed navigation filter.

To analyze whether the proposed error state Kalman filter is suited to bridge GNSS outages, GNSS outages of 30 s were simulated in postprocessing. The resulting time series for horizontal position and heading were then also compared to the reference trajectory.

During the field tests, the compost turner drove through two windrows at different speeds (0.2 m/s and 0.1 m/s). In both test runs, the horizontal positioning error was always below 10 cm, which is sufficient for the automatic steering of a compost turner for windrow composting. The mean error was 3.2 cm for the first test and 2.7 cm for the second test run. The maximum heading error was 1.3°, with the mean error being −0.4° for the first and −0.3° for the second test run.

During the simulated GNSS outages of 30 s, only the dynamic model which uses rotary encoder readings and a yaw rate measured by the IMU was used to estimate the position and attitude of the vehicle. During the simulated outages, the horizontal position still showed sub-decimetre accuracy. The mean horizontal positioning errors (3.0 cm for the first test run and 2.6 cm for the second test run) are in a similar range to the mean errors without GNSS outages. When GNSS outages were simulated, the heading deviated stronger from the reference heading than without GNSS outages (maximum deviation: 2.7°).

The first results of the field tests suggest that the proposed error state Kalman filter is suited to bridge GNSS outages of 30 s. The horizontal positioning accuracy of less than 10 cm after 30 s without GNSS is sufficient for the automatic steering of compost turners in windrow composting.

However, there are still limitations and further investigations are necessary before compost turners can be steered fully automatically. This study only investigated straight paths; to test and evaluate slip estimation for tracked vehicles properly, curved paths are necessary. Moreover, a vision-based approach is necessary if the compost turner should drive autonomously.

For future work, the achievable accuracies for position and heading with the proposed filter shall also be investigated for curved paths. Furthermore, a stereo camera and a precise 3D map of the compositing site will be added to the error state Kalman filter. By matching the 3D point cloud observed by the stereo camera to the map of the site, we hope to reduce position drifts during periods where no GNSS is available so that longer GNSS outages can be bridged.

## Figures and Tables

**Table 1:** Selected sensors used in the navigation algorithm.

Type	Model	Specification
GNSS	Alberding A12-RTK	Geodetic dual-antenna receiver + network RTK
IMU	XSens MTI-G-710	Mid-range MEMS IMU
Odometry	Atech AC-X	Motor inverter + rotary encoder for feedback

**Table 2:** Specifications of the used total stations for the reference trajectory. The accuracies for angle and distance measurements are taken from the manufacturer’s data sheets. The accuracy of the distance measurement listed in the table refers to the tracking mode.

Model	σ Angle Measurement	σ Distance Measurement
Leica Nova MS 60	$1^{\prime \prime }$ (0.3 mgon)	1 mm + 1.5 ppm
Leica TCRA1201	$1^{\prime \prime }$ (0.3 mgon)	3 mm + 1.5 ppm

**Table 3:** Mean deviation of the filtered solution from the reference trajectory for both test runs and standard deviation.

Deviation	Test Run 1	Test Run 2
North	0.8 ± 2.6 cm	−2.4 ± 1.8 cm
East	−1.6 ± 1.7 cm	1.0 ± 1.2 cm
Horizontal	3.2 ± 1.5 cm	2.7 ± 2.0 cm
Heading	−0.4 ± 0.4°	−0.3 ± 0.3°

**Table 4:** Mean deviation of the filtered solution from the reference trajectory with simulated GNSS outages of 30 for both test runs and standard deviation.

Deviation	Test Run 1	Test Run 2
North	−0.7 ± 2.4 cm	−1.9 ± 1.9 cm
East	−1.2 ± 1.8 cm	0.6 ± 1.5 cm
Horizontal	3.0 ± 1.3 cm	2.6 ± 1.6 cm
Heading	−0.8 ± 0.7°	−0.2 ± 0.6°

## Data Availability

Not applicable.

## Abbreviations

The following abbreviations are used in this manuscript:

EKF	Extended Kalman Filter
GNSS	Global Navigation Satellite Systems
IMU	Inertial Measurement Unit
INS	Inertial Navigation System
NED	North-East-Down
MEMS	Microelectromechanical systems
ROS	Robot Operating System
RTK	Real-Time Kinematic
